# Differences in Meiotic Recombination Rates in Childhood Acute Lymphoblastic Leukemia at an MHC Class II Hotspot Close to Disease Associated Haplotypes

**DOI:** 10.1371/journal.pone.0100480

**Published:** 2014-06-24

**Authors:** Pamela Thompson, Kevin Urayama, Jie Zheng, Peng Yang, Matt Ford, Patricia Buffler, Anand Chokkalingam, Tracy Lightfoot, Malcolm Taylor

**Affiliations:** 1 Paediatric & Familial Cancer Research Group, Institute of Cancer Sciences, University of Manchester, St Mary's Hospital, Manchester, United Kingdom; 2 School of Public Health, University of California, Berkeley, Berkeley, California, United States of America; 3 Department of Human Genetics and Disease Diversity, Tokyo Medical and Dental University, Tokyo, Japan; 4 School of Computer Engineering, Nanyang Technological University, Singapore; 5 Genome Institute of Singapore, A*STAR (Agency for Science, Technology, and Research), Biopolis, Singapore; 6 Data Analytics Department, Institute for Infocomm Research, A*STAR, Singapore; 7 Research Computing Services, Faculty of Medical and Human Sciences, University of Manchester, Manchester, United Kingdom; 8 University of York, Heslington, York, United Kingdom; 9 Independent Researcher, Handforth, Cheshire, United Kingdom; Mayo Clinic, United States of America

## Abstract

Childhood Acute Lymphoblastic Leukemia (ALL) is a malignant lymphoid disease of which B-cell precursor- (BCP) and T-cell- (T) ALL are subtypes. The role of alleles encoded by major histocompatibility loci (MHC) have been examined in a number of previous studies and results indicating weak, multi-allele associations between the *HLA-DPB1* locus and BCP-ALL suggested a role for immunosusceptibility and possibly infection. Two independent SNP association studies of ALL identified loci approximately 37 kb from one another and flanking a strong meiotic recombination hotspot (*DNA3*), adjacent to *HLA-DOA* and centromeric of *HLA-DPB1*. To determine the relationship between this observation and *HLA-DPB1* associations, we constructed high density SNP haplotypes of the 316 kb region from *HLA-DMB* to *COL11A2* in childhood ALL and controls using a UK GWAS data subset and the software PHASE. Of four haplotype blocks identified, predicted haplotypes in Block 1 (centromeric of *DNA3*) differed significantly between BCP-ALL and controls (P = 0.002) and in Block 4 (including *HLA-DPB1*) between T-ALL and controls (P = 0.049). Of specific common (>5%) haplotypes in Block 1, two were less frequent in BCP-ALL, and in Block 4 a single haplotype was more frequent in T-ALL, compared to controls. Unexpectedly, we also observed apparent differences in ancestral meiotic recombination rates at *DNA3*, with BCP-ALL showing increased and T-ALL decreased levels compared to controls. *In silico* analysis using LDsplit sotware indicated that recombination rates at *DNA3* are influenced by flanking loci, including SNPs identified in childhood ALL association studies. The observed differences in rates of meiotic recombination at this hotspot, and potentially others, may be a characteristic of childhood leukemia and contribute to disease susceptibility, alternatively they may reflect interactions between ALL-associated haplotypes in this region.

## Introduction

Acute lymphoblastic leukemia (ALL) is the most common malignant disease in children under 16 years of age in socio-economically developed countries including the UK [1]. Of the two lymphoid lineages, B-cell ALL comprises 85%, and T-cell ALL approximately 15% of cases; common or B-cell precursor ALL (BCP-ALL) is the most frequent ALL subtype overall. Whilst childhood ALL is predominantly a sporadic disease, a small fraction of cases arise in association with certain rare heritable or congenital genome instability disorders. Genome wide association studies (GWAS) of sporadic ALL have, however, identified single nucleotide polymorphisms (SNPs) in linkage disequilibrium (LD) with 4 genes (*IKZF1*, *ARID5B*, *CEBPE*, *CDKN2A*) [2], [3], [4] having roles in B-Cell development [5], [6], [7], [8]. A recent GWAS meta-analysis also identified two further SNPs tagging the genes *PIP4K2A* and *GATA3* as associated with specific BCP-ALL subtypes [9].

Although epidemiological studies have provided indirect support for an infectious etiology of BCP-ALL (reviewed in [10]), no causative agent has yet been identified. Clues provided by evidence that susceptibility to mouse retroviral ALL is linked to the MHC [11], and that certain human leukocyte antigen (HLA) alleles are associated with susceptibility to specific infections (reviewed in [12]), have encouraged the search for HLA allele associations with childhood ALL as a proxy for infection [13], [14], [15], [16], [17]. Previous studies of the *HLA-DPB1* locus which identified multiple, though weak, allele associations with ALL suggested a role for common antigenic peptide binding pockets in susceptibility. Interactions were identified between the DP1 supertype and proxies for delayed immune exposure in early life, providing further support for HLA-DP function in susceptibility to childhood BCP-ALL [14].

In contrast to these findings, recent GWAS analyses failed to detect SNPs strongly linked to HLA in childhood ALL. However, a modest (non-significant) association with the SNP rs3135034, approximately 97 kb centromeric of *HLA-DPB1*, was reported in a UK GWAS [18]. Furthermore, an independent SNP analysis of the extended MHC (xMHC) in a California ALL study revealed a significant association with rs9296068, located approximately 60 kb centromeric of *HLA-DPB1* [19]. The SNPs identified in these two studies are in close proximity to each other (∼37 kb apart) and flank the *HLA-DOA* locus.

In light of these findings, we considered the possibility that previous associations with *HLA-DPB1* alleles might be explained by linkage disequilibrium (LD) with a locus (or loci) in the vicinity of *HLA-DOA*. Our reasoning is based on evidence that the *HLA-DPB1* to *HLA-DOA* interval lies in a region of small haplotype blocks interspersed with pockets of LD breakdown, signifying hotspots of meiotic recombination [20]. The two BCP-ALL associated SNPs occur close to three recombination hotspots, of which *DNA3* is the most intense, leading us to hypothesize that LD breakdown between a putative ALL locus and *HLA-DPB1* could explain the weak allele associations and SNP results. Haplotype associations, LD breakdown and ancestral recombination rates in populations can be detected using estimation-based statistics [21]. Here, we applied these methods to the study of a 316 Kb region from *HLA-DMB* to *COL11A2* encompassing *HLA-DPB1* and *HLA-DOA* and the ALL associated SNPs.

## Results

### Haplotype frequencies in the *HLA-DMB* to *COL11A2* region in childhood ALL

In view of our results suggesting weak associations between certain *HLA-DPB1* alleles and childhood BCP-ALL [14], [16], [22], recent evidence [18] that an MHC SNP most strongly associated with childhood ALL (rs3135034) is approximately 97 kb telomeric of *HLA-DPB1*, and a significant association of ALL cases in the Northern California Childhood Leukemia Study (NCCLS) with rs9296068, located approximately 60 kb telomeric from *HLA-DPB1,* we considered that these SNPs might be in LD with BCP-ALL-associated *HLA-DPB1* alleles.

**Figure 1:**
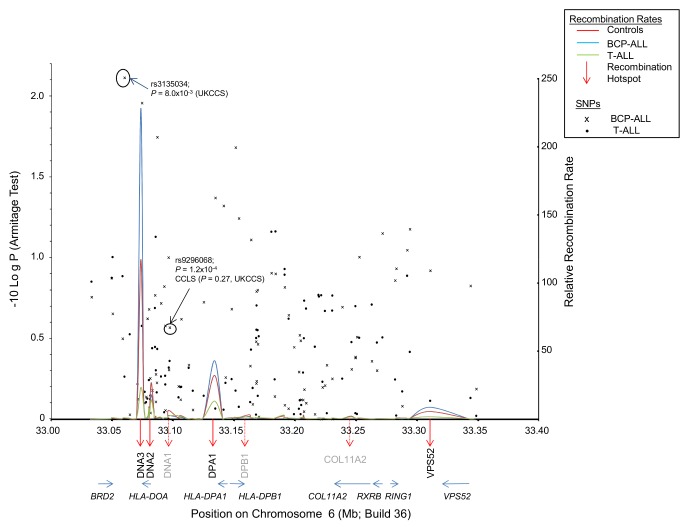
Relative recombination rates (right Y axis) at *DNA3* and flanking hotspots in Controls (red line), BCP-ALL cases (blue line), and T-ALL cases (green line), estimated using PHASE plotted over –Log P values (linear regression; left Y axis) for SNP associations. SNPs identified in array-based association studies of childhood ALL, rs3135034 (UKCCS [18]) and rs9296068 (CCLS)[19] are indicated. Recombination at *DNA3* is significantly higher in BCP-ALL cases than controls (P = 0.02), and significantly lower in T-ALL cases (P = 0.048). There is no significant difference at flanking hotspots.

We first examined the association of rs3135034 and rs9296068 with a subset of the ALL cases included in the UK GWAS [18] that identified rs3135034 in the context of childhood ALL. We compared the frequencies of 92 SNPs covering a ∼316 kb region of chromosome 6 (GRCh37, Chr6:32924583–33240505), in BCP-ALL and T-ALL cases and controls (N = 447, 44, and 2699, respectively). This region includes the *HLA-DOA* and *HLA-DPB1* loci, as well as rs3135034 and rs9296068. We confirmed the weak association between rs3135034 and BCP-ALL (uncorrected P = 5.4×10^−3^), but found no evidence of association of this SNP with T-ALL (P = 0.6), or an association of rs9296068 with either ALL subtype (P>0.3)(Figure 1; Table S1).

Using PHASE v2.1.1 [21], [23], [24], [25], we next determined the LD structure of the 316 kb region to identify haplotype blocks. Four haplotype blocks, separated by three recombination hotspots (predicted recombination rate ≥10x the background rate across the region, ρ [25], in at least one sample group) were defined: Block 1, 37.8 kb, with seven SNPs rs209474 - rs206767 (including rs3135034); Block 2, 4.4 kb, with three SNPs, rs172274 - rs3128931; Block 3, 40.1 kb, with 17 SNPs, rs86567 - rs7774158 (including rs9296068); and Block 4, 159.7 kb with 54 SNPs, rs3077 - rs213212 (including *HLA-DPB1*)(Table S1).

Case-control comparisons of overall association of variation within each haplotype block between case groups and controls (1000 permutations) revealed significant differences for BCP-ALL in Block 1 (P = 0.002) and T-ALL in Block 4 (P = 0.049), but no global difference in haplotype frequencies between either case group and controls for haplotype blocks 2 or 3 (P>0.097) (Table 1). Therefore, we compared specific haplotype frequencies in cases and controls using only haplotypes with frequencies >5% in at least one group (BCP-ALL, T-ALL, or controls) in haplotype Blocks 1 and 4. In Block 1, seven control and six case haplotypes had frequencies >5%; two haplotypes (1-C and 1-E) were significantly less frequent in BCP-ALL than controls (7.04% vs. 9.91% and 7.42% vs. 9.72%, respectively; uncorrected P<0.01; Table 2). Haplotype 1-C (P = 0.003) was the only common haplotype >5% to contain the minor allele of rs3135034, consistent with previous findings [2] and was the only one of these specific haplotype associations to withstand correction for multiple testing (Benjamini and Hochberg FDR; α = 0.0036). Haplotype block 4, encompassing the *HLA-DPA1* and *HLA-DPB1* loci, consisted of seven haplotypes >5%. Of these only one (4-B) was more frequent in T-ALL cases (18.14%) than controls (11.52%; uncorrected P = 0.04). PHASE analysis predicted that HLA-DPB1*06∶01, which we reported is associated with childhood ALL [13], would be carried on this haplotype. Ninety per cent of ALL cases with this haplotype were positive for DPB1*06∶01 (Table 2), consistent with our earlier finding that HLA-DPB1*06∶01 is significantly associated with T-ALL (Odds ratio, 95% confidence interval 10.0, 3.3–30.2; [13]), but not supportive of an association of this allele with BCP-ALL. In addition, the 4-G haplotype was marginally less common among BCP-ALL cases than controls (3.36 vs 4.86%; P = 0.03). Eighty five per cent of cases positive for the HLA-DPB1*01∶01 allele were predicted by PHASE predicts to carry haplotype 4-G (Table 2).

**Table 1:** Overall comparison of haplotype frequencies at four blocks in the MHC Class II region between childhood ALL cases and controls.

Haplotype Block	*P*, BCP-ALL Cases v Controls*	*P*, T-ALL Cases v Controls*
**1**	**0.002**	0.534
**2**	0.230	0.442
**3**	0.097	0.501
**4**	0.58	**0.049**

*P values were calculated by permutation testing (1000 permutations) within PHASE. Bold indicates P<0.05.

**Table 2:** Frequencies of specific haplotypes in blocks 1 and 4 in Controls, BCP-ALL and T-ALL.

Haplotype ID	Associated *HLA-DPB1* allele(s)	Control av. Freq (n = 2699)	BCP-ALL av. Freq (n = 447)	BCP-ALL, P	T-ALL av. Freq (n = 44)	T-ALL, P
**1-A**		27.05%	28.24%	0.2645	21.07%	0.1528
**1-B**		25.15%	27.17%	0.1063	32.86%	0.0679
**1-C**		9.91%	7.04%	0.0032*	7.88%	0.3481
**1-D**		9.90%	11.58%	0.0690	14.04%	0.1835
**1-E**		9.72%	7.42%	**0.0135**	6.71%	0.2386
**1-F**		9.03%	8.84%	0.4586	9.09%	0.6016
**1-G**		5.55%	4.77%	0.2040	4.56%	0.4567
**4-A**	*04∶01 (62%)	25.75%	28.15%	0.0702	20.58%	0.1572
**4-B**	*06∶01 (90%) *03∶01 (85%)	11.52%	11.14%	0.4104	18.14%	**0.0404**
**4-C**	*04∶02 (52%) *04∶01 (2%)	8.03%	9.04%	0.1590	12.98%	0.1127
**4-D**	*02∶01 (8%) *04∶01 (14%)	7.65%	8.39%	0.2561	6.90%	0.4868
**4-E**	*02∶01 (55%) *02∶02 (88%) *04∶02 (16%) *04∶01 (1%)	7.38%	8.66%	0.1046	6.75%	0.5235
**4-F**	*04∶01 (10%)	5.36%	4.43%	0.1553	4.58%	0.4901
**4-G**	*01∶01 (85%)	4.86%	3.36%	**0.0261**	8.39%	0.1385

P values are the results of two-tailed Fisher's Exact tests comparing control with either BCP-ALL or T-ALL frequency; bold format indicates uncorrected P<0.05. *P withstands Benjamini and Hochberg FDR correction for 14 haplotypes (α = 0.0036). *HLA-DPB1* alleles predicted by PHASE to be carried on each Block 4 haplotype are also shown (percentages in brackets indicate the proportion of each *DPB1* allele predicted to be carried on the haplotype.

### Case-control comparison of recombination rates

We observed that the number of case haplotypes in each block differed from the number in controls when we used control groups containing the same number of individuals as each case group. For BCP-ALL vs controls (n = 447 in each group) 1040 vs. 991 haplotypes, respectively, were predicted whereas for T-ALL vs controls (n = 44 in each group) 250 vs. 527 haplotypes, respectively, were predicted (Table S2). We suspected that these differences could be due to different recombination rates across the 92 SNP interval in cases compared with controls. This is supported by the observation that the T-ALL group showed a lower (23 x), whilst BCP-ALL had a much higher (228 x), recombination rate at the strongest hotspot, *DNA3*, compared with controls (117 x background) (Figure 1).

**Figure 2:**
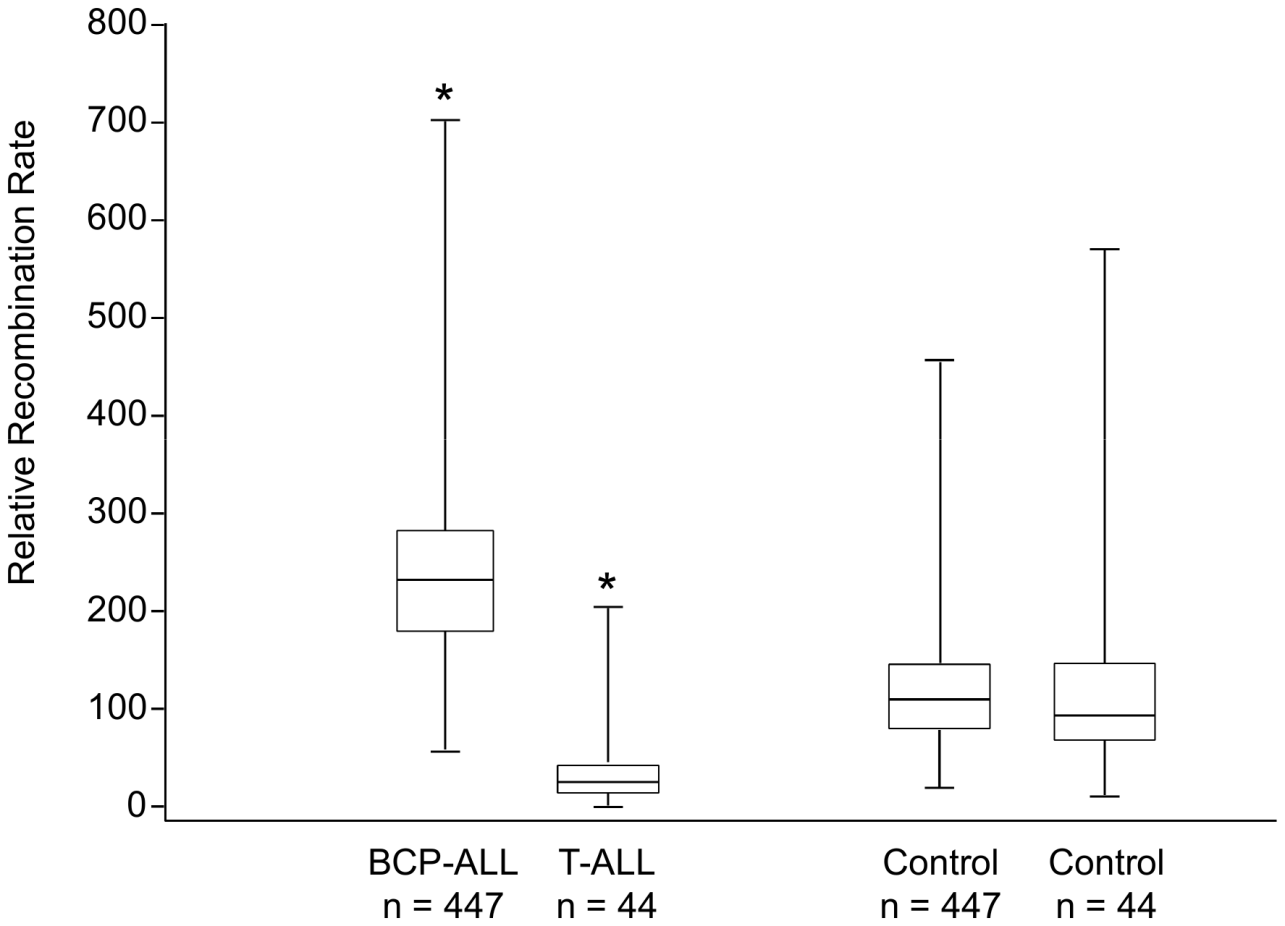
Box and whisker plots illustrating medians, quartiles, and ranges of recombination rates estimated using PHASE software between rs176248 and rs206767 (*DNA3* hotspot) in childhood leukemia cases and controls. Sample size (n = 447 and n = 44) of randomly selected controls did not significantly affect recombination rate estimates. Relative recombination rates (y- axis) are significantly higher in BCP-ALL (n = 447; P = 0.02) and significantly lower in T-ALL (n = 44; P = 0.048) cases than controls.

Since the number of PHASE-predicted haplotypes in a group (here cases or controls) is not only a function of the recombination rate but also of the number of individuals included in the group, we tested different numbers of randomly selected controls as comparators. We found that where the number of controls (447 or 44) matched the number of cases in each ALL sub-type, this did not significantly affect recombination rate estimates (Figure 2).

We carried out permutation tests to determine if the observed differences in case-control recombination rates were significant, and found that differences in recombination rates at the *DNA3* hotspot were significant (BCP-ALL, P = 0.02; T-ALL, P = 0.048). Although case-control differences in recombination rates detected by PHASE were observed at three other hotspots (*DNA2*, *DPA1*, and *VPS52*) they were not significant (P>0.18); however, the rates at *DPA1* and *VPS52* showed the same trend as *DNA3* (ie BCP-ALL > Control > T-ALL). Median recombination rates at *DNA2* were similar in all three study groups though marginally higher in controls than in BCP-ALL and T-ALL (Figure 1). Although the previously identified *‘DNA1*’ locus [20] did not meet our definition of a recombination hotspot (ie. recombination rate >10 x background), there was evidence of increased recombination at this locus (between rs3128931 and rs1044429) with similar rates of 8.0, 5.0, and 9.3 x background in T-ALL, BCP-ALL, and Controls, respectively.

As polymorphisms at the gene *PRDM9*, which encodes a meiosis specifc histone methyltransferase, are known to specify hotspot location and intensity [26], we plotted publically available data from an Icelandic population [27] to investigate whether genotype at *PRDM9* is likely to influence recombination rates in the region of chromosome 6 investigated in this study. The results indicated that recombination rates, including those at *DNA3*, are likely to be strongly affected by the numbers of zinc fingers in PRDM9 (Figure S1).

### Association of SNPs flanking *DNA3* with hotspot intensity

**Figure 3:**
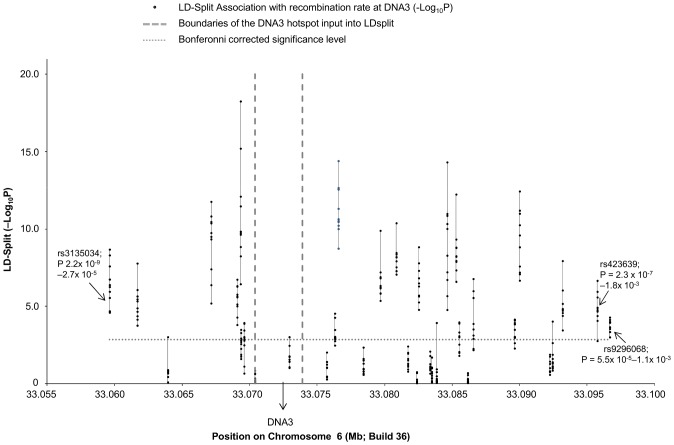
Results of LDsplit analysis for association of SNPs flanking *DNA3* with hotspot intensity. For each SNP included in the HAPMAP-CEU phased haplotypes (Table S3), ten estimates of -Log10P values for association with hotspot intensity are plotted (black spots joined by lines). P values of SNPs identified in association studies of childhood ALL are indicated. The boundaries of the *DNA3* hotspot used for the analysis are indicated by vertical dashed lines and the corrected P value cut-off is shown by a horizontal dotted line.

Recent work using the LDsplit program [28], [29] to analyze population genetic data has shown that *cis*-acting loci are associated with recombination hotspot activity. This confirms results obtained using traditional sperm-typing methodology [20]. In an effort to identify these *cis*-acting loci affecting recombination rates at *DNA3*, we used LDsplit to compare recombination rates associated with SNP alleles at flanking polymorphic sites. Thirty-five tag SNPs (MAF>0.07; Table S3.) in the region (chr6:33058028–33097923; GRCh36) were identified in the European (CEU) population, using the algorithm Tagger (pairwiseTagging)[30] and phased haplotypes, available from the HapMap website, were used as input data for LDsplit. LDsplit did not identify any single SNP as having a dominant *cis*-influence over recombination rates at *DNA3*. However, using a Bonferroni corrected P value of 1.4×10^−3^, 13 of the 35 tag SNPs were associated with levels of recombination at *DNA3* (P values for all 10 replicate analyses <1.4×10^−3^). The SNPs identified in the UKCCS (rs3135034) and NCCLS (rs9296068) were among those linked to hotspot intensity, with P values ranging from 2.2×10^−9^–2.7×10^−5^ and 5.5×10^−5^–1.1×10^−3^, respectively (Figure 3.). rs3135034 is ∼14 kb centromeric and rs9296068 is ∼23 kb telomeric from the centre of *DNA3*, with the major and minor alleles at these loci, respectively, being associated with increased hotspot intensity. These results suggest that several *cis*-acting loci in this region, including those identified in studies of childhood ALL, may influence recombination rates at *DNA3*.

## Discussion

Haplotype analysis in this study differed from that of both Hosking et al [18] and Urayama et al [19] as we used the PHASE algorithm (Stephens et al. 2001; Li and Stephens 2003) which has the advantage of taking into account similarities in haplotypes, rather than simply comparing frequencies of identical haplotypes, so that, even if every individual case and control carried a different combination of alleles, if they are more similar within than between groups, this can be detected. Haplotype associations identified here using this method are of a similar magnitude to those previously reported for the UKCCS data in single SNP analyses [18]; however, rather than a simple association of the major allele of rs3135034 with leukemia risk, our data suggests the possible presence of two haplotypes protective for leukemia, one carrying the major, and the other being the only common haplotype carrying the minor, allele at rs3135034. This would translate into an uncorrected odds ratio, 95% confidence interval of 1.26, 1.06–1.51 (P = 0.004) for BCP-ALL development in patients carrying the major allele on one of the remaining five common haplotypes. This haplotype block spans the *BRD2* locus, which has been associated with Juvenille Epilepsy, and is not an obvious candidate risk factor for ALL, despite its relatively wide expression pattern [31]. Taken in combination with published data from the California Childhood Leukemia study [19], and the results of LDsplit analyses which indicate multiple cis-acting sequences, including loci identified in the SNP association studies, contributing to recombination at *DNA3*, this is suggestive of an association of these haplotypes and recombination events affecting *HLA-DOA*, with BCP-ALL. *HLA-DOA* encodes one component of the heterodimeric HLA-DO molecule, a non-classical HLA molecule which contributes to the antigen-recognition repertoire of CD4+ T-cells through influencing intracellular peptide loading by HLA-DM by acting as a substrate mimic [32]. HLA-DO expression is restricted to professional antigen presenting cells, including B-cells and those of the thymic epithelium [33]. The modulation of MHC II antigen presentation is of vital importance in immune regulation, providing a potential link between genetic susceptibility to childhood ALL and postulated immune system dysregulation [34].

The data presented in this paper provide evidence of alterations in ancestral recombination rates in the MHC Class II region in childhood ALL populations compared to ethnically matched controls, with lower and higher rates than controls at the *DNA3* hotspot in populations of children who developed T-ALL and BCP-ALL, respectively. Our results are consistent with those of recent family studies, which uncovered a substantial overrepresentation of rare *PRDM9* alleles in the parents of children with BCP-ALL compared to ethnically matched controls in two North American populations [35]. Furthermore, our data indicate that differences in recombination rates may be detectable by analysis of Western European population genetic data, representing ancestral meiotic crossover events through many generations, suggesting that some populations carrying these alleles are at increased risk for development of childhood leukemias. However, as discussed by Hussin et al., [35], the relatively high frequency among Africans [26], [36] of the rare *PRDM9* alleles associated with childhood ALL in Caucasian populations, along with a proportionally low incidence of childhood leukemias among African-Americans [37] indicates that variant alleles of *PRDM9* are insufficient to predispose to leukemia and suggestive of further contributory parental genetic background factors. Moreover, another plausible interpretation of our results is that recombination rates in ancestors of ALL patients are the same as those of ancestors of controls, with the observed changes being due to association of disease with interacting alleles in different haploytpe blocks, such that associated allele combinations are selected for in the disease groups, leading to the appearance of increased recombination. Given the recent reports of an association of rare *PRDM9* alleles with ALL [35], we favour the hypothesis that altered recombination rates are also associated with this disease. However, further work to distinguish between these possibilities will be required.

Disease associations coinciding with recombination hotspots are rare, due to the statistical methods employed in genetic susceptibility studies; indeed it has been noted that etiological variants within a recombination hot spot may be impossible to identify using standard association strategies [38]. However, a schizophrenia locus within a recombination hotspot has been described [39] and a Type 1 Diabetes susceptibility locus has also been mapped in mouse [40]. In both studies, mechanisms of locus-specific disease etiology effects were shown to be dependent on sequence variation mediated by recombination events. Given the identification in two studies of childhood ALL of SNPs flanking a recombination hotspot coinciding with the *HLA-DOA* coding sequence, and the observed apparent variations in recombination rates at this locus among our study groups, we speculate that similar recombination mechanisms may functionally influence *HLA-DOA* sequence in childhood leukemia, although this will require experimental verification. Although a previous study of sperm recombinants indicated that recombination events at the *DNA3* hotspot invariably resulted in fully reciprocal crossovers [20], it is not clear if this will also be true of female recombination, or whether the unusual distribution of *PRDM9* genotypes among the parents of children with ALL [35] may influence the nature of crossovers at *DNA3*.

Among our patient samples, the 4-G haplotype was moderately less frequent among BCP-ALL cases than controls (3.36 vs 4.86%; P = 0.03); this is the predominant carrier of the *HLA-DPB1* allele DPB1*01∶01 (in 85% of ALL cases typed DPB1*01∶01, PHASE predicted that the allele would be carried on the 4-G haplotype) (Table 2). HLA-DPB1*01∶01 has previously been reported to be underrepresented among UKCCS ALL patients [41] and was recently reported to be associated with an increased risk for ALL in the NCCLS [14]. In addition, our results are suggestive that this haplotype may be overrepresented among T-ALL patients in the UK population (8.39%; P = 0.1). As childhood T-ALL is relatively rare compared to BCP-ALL, this study was underpowered to detect associations of the expected magnitude; however, our results strongly support a specific association of T-ALL with haplotypes carrying the HLA-DPB1*0601 locus. Further investigations of combined data from different studies will be highly desirable for T-ALL.

In conclusion, this study provides evidence of potentially significant population level differences in meiotic recombination at the *HLA-DOA* locus in BCP- and T-ALL, with increased and decreased activity, respectively. These findings will require replication in other sample sets and at other hotspots, and as discussed above, may alternatively reflect interactions between disease associated loci in this region. However, the intensity of this hotspot is strongly influenced by variation at *PRDM9* (Figure S1) and our data should be interpreted in the context of the recent discovery of substantial overrepresentation of rare PRDM9 alleles in parents of children with ALL, combined with unusual recombination patterns [35]. Hence our results suggest that differences in hotspot magnitude, in addition to location, can be observed, and that these are detectable by population-based methods, raising the possibility of genetically defined sub-populations with increased susceptibility to childhood leukemia.

## Materials and Methods

### Patients and Controls

The UKCCS was a population-based case control study of childhood malignant disease, carried out in the UK between 1992 and 1998. Case diagnoses were classified as previously described [42] as B cell precursor (BCP-) ALL, and T-ALL and validated by the UKCCS data centre at the University of York, UK. We used SNP data generated from cases and controls as part of a previously described GWAS [2]. Data were available from 447 cases of BCP-ALL, 44 cases of T-ALL, and 2699 control (Wellcome Trust Case Control 1958 Birth Cohort) samples. Only data from individuals confirmed by principal component analysis to be of Western European ancestry were included in the analyses [2].

### Comparison of Case and Control allele and haplotype frequencies

Single SNP allele frequencies were compared using SNP & Variation Suite v7.5.5 (Golden Helix, Inc., Bozeman, MT, www.goldenhelix.com), using linear regression analysis (additive model), with correction for stratification by principal component analysis (eigenvalues).

To identify haplotype blocks, case and control SNP data from all 92 observed loci in the region chr6:32924583–33240505 (GRCh37) genotyped in the UKCCS GWAS were input into the PHASE (v2.1.1) program [21], [23], [24], [25] and run using the flags -MR, -S*, and -X10; where –MR specifies the use of the recombination model for estimation of haplotypes (this takes account of the presence of recombination hotspots), -X10 sets the number of iterations performed in the final run to be 10x greater than the default (recommended by the software authors to increase confidence in output data, particularly where recombination rates are estimated), and –S the specification of a randomly generated seed (*). The analysis was performed four times, using a different seed each time, for each set of cases and controls, also to increase confidence in output data, in accordance with the recommendations of the software authors.

Haplotype blocks were defined as contiguous groups of SNPs where recombination rates did not exceed 5x background rate. Of the 92 SNPs those excluded from haplotype blocks by these criteria were rs176248, rs2395300, rs1044429, rs2581, rs2284191, rs2395309, rs213220, rs213199, and rs464921, which either defined recombination hotspots, or were isolated at the telomeric end of the region under investigation (Table S1).

To compare overall haplotype frequencies between case and control groups input files consisting of both cases and controls (indicated by their identifiers) were run in PHASE v2.1.1 [21], [23], [24], [25] using the flag –c1000 to specify comparison between the two groups using 1000 permutations. The statistical significance of differences between case and control frequencies of specific haplotypes in each block was calculated using the Fisher's Exact test.

For determination of associations between individual haplotypes and *HLA-DPB1* alleles, coded *HLA-DPB1* types [13] were included in PHASE input data (between rs3135021 and rs9277535) before calculation of haplotypes (N.B. This was not done for case-control haplotype comparison analysis as *HLA-DPB1* types are not available for the control samples) and the percentage of each *HLA-DPB1* allele predicted to occur in a specific haplotype (frequency occurring on haplotype/frequency in the population) calculated. The number of samples with available *HLA-DPB1* types included in this analysis was 453.

### Estimation of the location and strength of Recombination Hotspots

Recombination hotspot locations (defined as relative recombination rate >10x background rate across the region, ρ [25] in cases or controls, were identified from PHASE *.out_recom files, which also provided estimates of recombination rates between each pair of adjacent loci included. Each run generated 1000 estimates of recombination rates between adjacent SNPs and the results of 4 runs were combined to give estimated values based on the median of 4000 values generated for each pair of loci.

The significance of differences between predicted case and control recombination rates were tested by performing 250 permutation tests (a number chosen as the maximum practicable, given the computing time involved) using input data where case and control samples were randomly assigned pseudo-case or pseudo-control status. As for the actual case and control data, analyses were performed four times using different seeds. Medians of each set of 4000 values generated were calculated and the difference between these for 250 pseudo-case/pseudo-control pairs calculated for SNPs flanking hotspots defined by the initial PHASE analysis (rs176248 and rs206767; rs2395300 and rs176248; rs2581 and rs1044429; rs2395309 and rs375912).

### Association of hotpots with proximal SNPs

CEU-phased haplotypes (Utah residents with ancestry from northern and western Europe; HapMap Phase 3, release 2), available from the HapMap website, were used as input data for the program LDsplit, which was previously used to confirm the association of the SNP ‘FG11’ (rs416622; [43]) with recombination at *DNA2*. CEU phased haplotypes were used in preference to the case and control data generated in this study due to the higher density of SNPs included in the HapMap data, compared to our GWAS-generated information. Thirty-five tag SNPs (MAF>0.07; Table S2) were identified for the population CEU in the region (chr6:33058028–33097923; GRCh36), using the algorithm Tagger (pairwise Tagging)[30]. Ten estimates of P values for association of each SNP with recombination rates at *DNA3* were generated by LDsplit. For SNPs with MAF>0.1, these were generated using a different randomly selected 190 CEU haplotypes as input for each estimate, and for SNPs with MAF<0.1, all haplotypes which carried the minor allele were included in each replicate, combined with randomly selected haplotypes carrying the major allele to a total of 160. LDsplit parameters were set as follows: -iteration, 2000000; -burn, 5000; -sample, 200; -random permutations; 200.

## Supporting Information

Figure S1 **Recombination Rates in Icelandic Population Data According to PRDM9 Genotype**. Plots of male (A) and female (B) recombination rates (chr6:33,000,000–33,400,000) in Icelandic parent-child pairs stratified by PRDM9 ZnF variants (data obtained from supplementary tables associated with [27]). Red line, recombination rate with 12–13 ZnF motifs. Blue line, recombination rate with 14–15 ZnF motifs. There are two female specific hotspots in this region, one of which (COL11A2) is ablated in individuals with 14–15 ZnF motifs and the other (*DPB1*) is enhanced. Of three other visible hotspots (*DNA1-3*, *DPA1* and *VPS52*), which occur in both males and females, all are ablated in males carrying 14–15 ZnF motifs, whereas, although both the *DNA1–3* and *VPS52* hotspots are absent, there appears to be little effect of the presence of this variant on recombination at *DPA1* in females.
(DOCX)

Table S1 **Association tests for 92 single SNPs in the MHC Class II region from** ***HLA-DMB*** **to** ***COL11A2*** **and haplotype block definitions.** Results shown are P values of linear regression analysis (additive mode), corrected for stratification by principal component analysis using SNP and Variation Suite (v 7.7.5). SNPs identified in previous studies (rs3135034 and rs9296068) [18], [19] are highlighted in bold. SNPs included in haplotype blocks 1–4 are shaded.
(DOCX)

Table S2 **Haplotype Numbers Predicted by PHASE.** Average numbers of predicted haploytpes using matched numbers of cases and controls (BCP-ALL, 447; T-ALL, 44) based on output data from four replicate PHASE analyses using different seed values.
(DOCX)

Table S3 **Tagger Selected SNPs.** Thirty five SNPs, selected using Tagger [44] for haplotype analysis with LDsplit [28], showing chromosomal location and minor allele frequency (MAF) in the CEU population.
(DOCX)
