# Supplementary figures and images for: Differences in Meiotic Recombination Rates in Childhood Acute Lymphoblastic Leukemia at an MHC Class II Hotspot Close to Disease Associated Haplotypes
Source: PLoS One. 2014 Jun 24;9(6):e100480. doi: 10.1371/journal.pone.0100480 (PMC4069019; doi:10.1371/journal.pone.0100480)

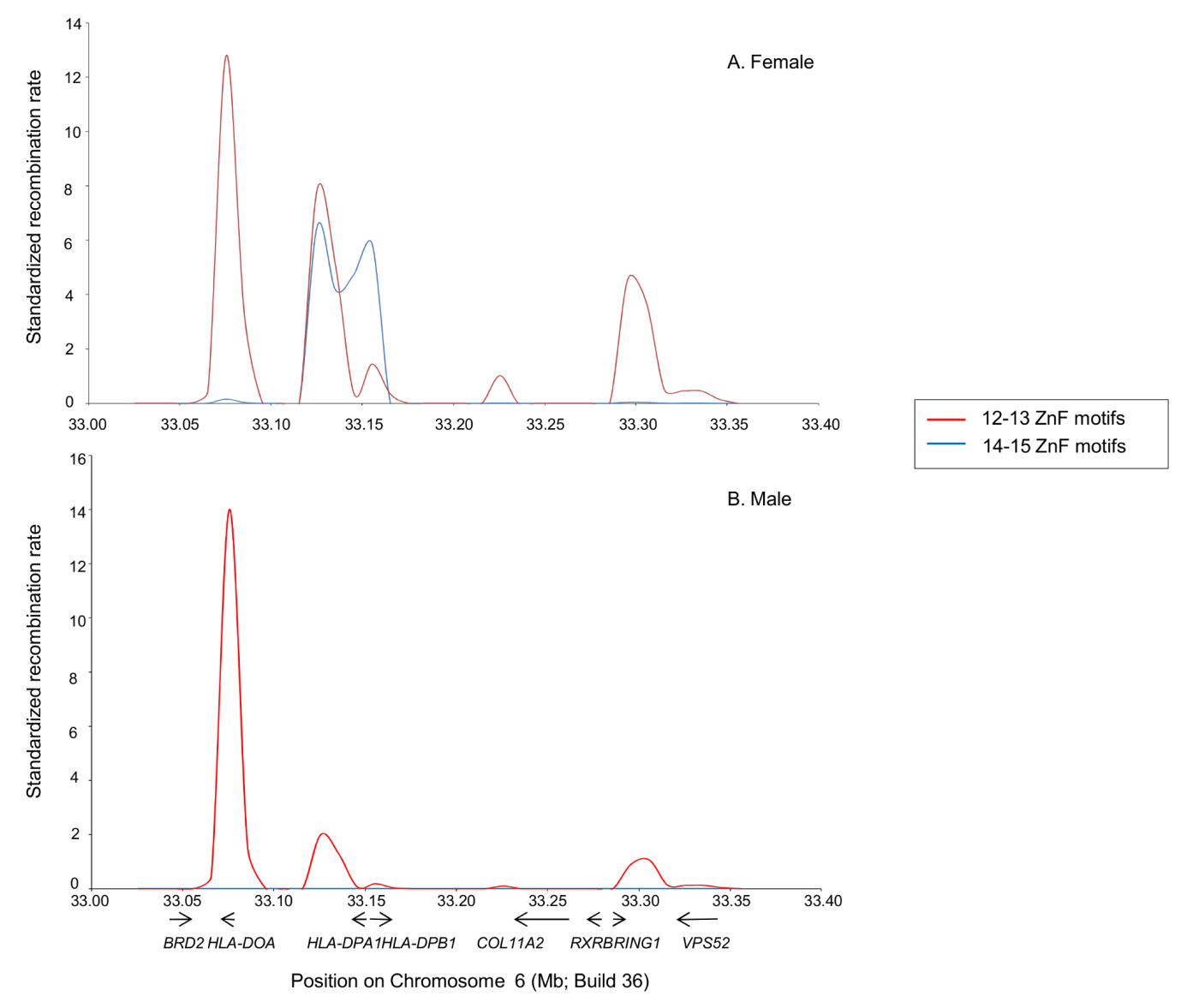


**Figure S1.** **Recombination Rates in Icelandic Population Data According to PRDM9 Genotype**

Supplement: Figure S1 — Recombination Rates in Icelandic Population Data According to PRDM9 Genotype. Plots of male (A) and female (B) recombination rates (chr6:33,000,000–33,400,000) in Icelandic parent-child pairs stratified by PRDM9 ZnF variants (data obtained from supplementary tables associated with [27]). Red line, recombination rate with 12–13 ZnF motifs. Blue line, recombination rate with 14–15 ZnF motifs. There are two female specific hotspots in this region, one of which (COL11A2) is ablated in individuals with 14–15 ZnF motifs and the other (DPB1) is enhanced. Of three other visible hotspots (DNA1-3, DPA1 and VPS52), which occur in both males and females, all are ablated in males carrying 14–15 ZnF motifs, whereas, although both the DNA1–3 and VPS52 hotspots are absent, there appears to be little effect of the presence of this variant on recombination at DPA1 in females. (DOCX) [file pone.0100480.s001.docx]
