# Supplementary material for: Differences in Meiotic Recombination Rates in Childhood Acute Lymphoblastic Leukemia at an MHC Class II Hotspot Close to Disease Associated Haplotypes
Source: PLoS One. 2014 Jun 24;9(6):e100480. doi: 10.1371/journal.pone.0100480 (PMC4069019; doi:10.1371/journal.pone.0100480)
Supplement: Table S1 — Association tests for 92 single SNPs in the MHC Class II region from HLA-DMB to COL11A2 and haplotype block definitions. Results shown are P values of linear regression analysis (additive mode), corrected for stratification by principal component analysis using SNP and Variation Suite (v 7.7.5). SNPs identified in previous studies (rs3135034 and rs9296068) [18], [19] are highlighted in bold. SNPs included in haplotype blocks 1–4 are shaded. (DOCX) [file pone.0100480.s002.docx]

**Table S1.** **Association tests for 92 single SNPs in the MHC Class II region from *HLA-DMB* to *COL11A2* and haplotype block definitions.**

|  |  |  |  |  | **BCP-ALL** | | **T-ALL** | |
| --- | --- | --- | --- | --- | --- | --- | --- | --- |
| **Marker ID** | **Chr6: Position**  **(GRCh36)** | **Hap Block** | **MAF** | **Allele Freq. (Controls)** | **Regression P** | **Allele Freq. (Cases)** | **Regression P** | **Allele Freq. (Cases)** |
| rs209474 | 33032562 | 1 | 0.37 | 0.37 | 3.13E-01 | 0.39 | 1.71E-01 | 0.44 |
| rs3130597 | 33049169 | 1 | 0.10 | 0.10 | 3.29E-01 | 0.12 | 8.54E-03 | 0.15 |
| rs620202 | 33049888 | 1 | 0.26 | 0.25 | 6.45E-01 | 0.27 | 1.34E-01 | 0.33 |
| rs565876 | 33058028 | 1 | 0.35 | 0.34 | 8.16E-01 | 0.36 | 1.65E-01 | 0.42 |
| **rs3135034** | **33059640** | 1 | **0.10** | **0.10** | **5.41E-03** | **0.07** | **6.18E-01** | **0.08** |
| rs188245 | 33063954 | 1 | 0.46 | 0.46 | 4.27E-01 | 0.46 | 7.23E-01 | 0.51 |
| rs206767 | 33070398 | 1 | 0.48 | 0.48 | 3.12E-01 | 0.49 | 3.19E-01 | 0.45 |
| rs176248 | 33073920 |  | 0.30 | 0.30 | 2.48E-01 | 0.26 | 4.00E-01 | 0.25 |
| rs2395300 | 33076254 |  | 0.30 | 0.30 | 4.87E-01 | 0.29 | 6.92E-01 | 0.32 |
| rs172274 | 33077435 | 2 | 0.31 | 0.32 | 5.89E-01 | 0.31 | 6.31E-01 | 0.33 |
| rs206762 | 33078428 | 2 | 0.45 | 0.45 | 5.71E-01 | 0.47 | 9.12E-01 | 0.47 |
| rs3128931 | 33079686 | 2 | 0.25 | 0.25 | 8.30E-01 | 0.23 | 6.15E-01 | 0.23 |
| rs1044429 | 33080620 |  | 0.11 | 0.11 | 4.38E-01 | 0.11 | 8.67E-01 | 0.13 |
| rs376892 | 33080865 |  | 0.29 | 0.29 | 1.44E-01 | 0.29 | 7.77E-01 | 0.27 |
| rs2581 | 33082379 |  | 0.46 | 0.46 | 2.54E-01 | 0.47 | 5.49E-01 | 0.41 |
| rs2284191 | 33084632 |  | 0.09 | 0.09 | 7.40E-01 | 0.09 | 8.61E-01 | 0.13 |
| rs86567 | 33084737 | 3 | 0.44 | 0.44 | 7.41E-01 | 0.46 | 3.33E-01 | 0.39 |
| rs403414 | 33085293 | 3 | 0.18 | 0.18 | 4.36E-01 | 0.18 | 6.44E-02 | 0.25 |
| rs381218 | 33085398 | 3 | 0.26 | 0.26 | 9.80E-01 | 0.27 | 2.82E-01 | 0.23 |
| rs6911639 | 33086156 | 3 | 0.22 | 0.22 | 7.74E-01 | 0.21 | 6.33E-01 | 0.20 |
| rs429916 | 33086565 | 3 | 0.08 | 0.09 | 2.72E-01 | 0.06 | 1.77E-01 | 0.11 |
| rs6457699 | 33089625 | 3 | 0.44 | 0.44 | 4.98E-01 | 0.42 | 7.51E-01 | 0.48 |
| rs9276994 | 33092233 | 3 | 0.35 | 0.36 | 6.85E-01 | 0.33 | 6.90E-01 | 0.35 |
| rs3130604 | 33093030 | 3 | 0.16 | 0.16 | 6.08E-01 | 0.18 | 9.29E-01 | 0.14 |
| rs1367731 | 33093177 | 3 | 0.18 | 0.18 | 1.52E-02 | 0.18 | 7.82E-01 | 0.16 |
| rs423639 | 33095752 | 3 | 0.08 | 0.08 | 1.28E-01 | 0.06 | 3.80E-01 | 0.08 |
| rs6457702 | 33096027 | 3 | 0.47 | 0.47 | 5.33E-02 | 0.48 | 6.40E-01 | 0.43 |
| **rs9296068** | **33096673** | 3 | **0.31** | **0.31** | **9.14E-01** | **0.30** | **3.00E-01** | **0.35** |
| rs6933546 | 33103992 | 3 | 0.33 | 0.33 | 9.68E-01 | 0.33 | 9.78E-01 | 0.35 |
| rs6920606 | 33105652 | 3 | 0.45 | 0.45 | 8.24E-01 | 0.45 | 8.83E-01 | 0.48 |
| rs9277027 | 33106216 | 3 | 0.26 | 0.26 | 5.08E-02 | 0.24 | 8.77E-01 | 0.28 |
| rs763469 | 33112365 | 3 | 0.17 | 0.16 | 2.81E-01 | 0.17 | 8.28E-01 | 0.14 |
| rs7774158 | 33115730 | 3 | 0.34 | 0.34 | 8.88E-01 | 0.34 | 8.93E-01 | 0.36 |
| rs375912 | 33124706 |  | 0.31 | 0.31 | 4.51E-02 | 0.29 | 9.05E-01 | 0.33 |
| rs2395309 | 33134224 |  | 0.17 | 0.18 | 6.76E-01 | 0.15 | 7.37E-01 | 0.17 |
| rs3077 | 33141000 | 4 | 0.17 | 0.18 | 6.72E-01 | 0.15 | 7.38E-01 | 0.17 |
| rs2301226 | 33142574 | 4 | 0.12 | 0.12 | 3.07E-01 | 0.13 | 7.44E-01 | 0.10 |
| rs9277341 | 33147603 | 4 | 0.30 | 0.30 | 2.25E-01 | 0.28 | 9.97E-01 | 0.27 |
| rs987870 | 33150858 | 4 | 0.14 | 0.14 | 6.22E-01 | 0.11 | 5.81E-01 | 0.16 |
| rs3135021 | 33153536 | 4 | 0.27 | 0.26 | 1.65E-02 | 0.29 | 5.77E-01 | 0.27 |
| rs9277535 | 33162839 | 4 | 0.25 | 0.25 | 2.92E-01 | 0.24 | 6.19E-01 | 0.26 |
| rs9277554 | 33163516 | 4 | 0.30 | 0.31 | 5.66E-01 | 0.28 | 6.00E-01 | 0.35 |
| rs9277565 | 33164875 | 4 | 0.21 | 0.21 | 1.75E-01 | 0.20 | 3.98E-01 | 0.23 |
| rs2281390 | 33167647 | 4 | 0.16 | 0.16 | 8.86E-01 | 0.17 | 1.55E-01 | 0.16 |
| rs2281389 | 33167774 | 4 | 0.17 | 0.17 | 8.40E-01 | 0.17 | 9.39E-01 | 0.22 |
| rs3128917 | 33167974 | 4 | 0.26 | 0.27 | 5.25E-01 | 0.24 | 9.69E-01 | 0.32 |
| rs2281388 | 33168096 | 4 | 0.02 | 0.02 | 2.85E-01 | 0.02 | 3.05E-01 | 0.00 |
| rs3117222 | 33168927 | 4 | 0.26 | 0.27 | 5.29E-01 | 0.24 | 9.70E-01 | 0.32 |
| rs3128918 | 33169076 | 4 | 0.16 | 0.16 | 9.81E-01 | 0.17 | 3.96E-01 | 0.17 |
| rs3130192 | 33169908 | 4 | 0.10 | 0.10 | 2.81E-01 | 0.10 | 4.77E-01 | 0.14 |
| rs2064478 | 33180244 | 4 | 0.23 | 0.24 | 9.34E-01 | 0.21 | 9.77E-02 | 0.32 |
| rs3130215 | 33182941 | 4 | 0.43 | 0.43 | 2.07E-02 | 0.43 | 6.25E-01 | 0.40 |
| rs3117230 | 33183613 | 4 | 0.23 | 0.24 | 9.57E-01 | 0.21 | 9.32E-02 | 0.32 |
| rs1810472 | 33191099 | 4 | 0.29 | 0.29 | 5.33E-01 | 0.27 | 4.28E-01 | 0.22 |
| rs2395349 | 33191112 | 4 | 0.19 | 0.19 | 6.10E-01 | 0.19 | 2.33E-01 | 0.13 |
| rs3117035 | 33194227 | 4 | 0.41 | 0.41 | 5.13E-01 | 0.41 | 4.23E-01 | 0.41 |
| rs1883414 | 33194426 | 4 | 0.31 | 0.32 | 4.13E-01 | 0.30 | 8.61E-01 | 0.38 |
| rs4713607 | 33198814 | 4 | 0.48 | 0.48 | 9.35E-01 | 0.47 | 1.41E-01 | 0.48 |
| rs3129274 | 33202847 | 4 | 0.33 | 0.33 | 7.04E-01 | 0.34 | 6.81E-02 | 0.34 |
| rs3117016 | 33203494 | 4 | 0.39 | 0.39 | 6.76E-01 | 0.37 | 5.58E-01 | 0.38 |
| rs3117008 | 33204252 | 4 | 0.48 | 0.48 | 7.63E-01 | 0.46 | 1.40E-01 | 0.48 |
| rs3117004 | 33204744 | 4 | 0.32 | 0.32 | 7.63E-01 | 0.31 | 9.47E-01 | 0.31 |
| rs6901221 | 33206254 | 4 | 0.16 | 0.16 | 9.87E-01 | 0.15 | 5.45E-01 | 0.18 |
| rs2294478 | 33206944 | 4 | 0.46 | 0.46 | 8.62E-01 | 0.46 | 8.27E-02 | 0.41 |
| rs2294472 | 33207188 | 4 | 0.38 | 0.38 | 1.76E-01 | 0.38 | 1.91E-01 | 0.31 |
| rs2395352 | 33208710 | 4 | 0.37 | 0.37 | 5.22E-01 | 0.38 | 2.77E-01 | 0.31 |
| rs4713610 | 33215933 | 4 | 0.16 | 0.16 | 7.89E-01 | 0.16 | 3.49E-01 | 0.13 |
| rs7754200 | 33218911 | 4 | 0.22 | 0.22 | 5.52E-01 | 0.22 | 6.63E-01 | 0.28 |
| rs3129234 | 33219325 | 4 | 0.22 | 0.22 | 6.48E-01 | 0.22 | 6.16E-01 | 0.28 |
| rs3129223 | 33221175 | 4 | 0.22 | 0.22 | 5.97E-01 | 0.22 | 6.32E-01 | 0.28 |
| rs1003979 | 33222149 | 4 | 0.44 | 0.44 | 3.09E-01 | 0.43 | 4.33E-01 | 0.41 |
| rs2395365 | 33224200 | 4 | 0.40 | 0.40 | 1.16E-01 | 0.41 | 9.61E-01 | 0.47 |
| rs721394 | 33225796 | 4 | 0.18 | 0.18 | 2.78E-01 | 0.19 | 6.74E-01 | 0.18 |
| rs756441 | 33230149 | 4 | 0.40 | 0.40 | 9.61E-02 | 0.41 | 9.59E-01 | 0.47 |
| rs756440 | 33230309 | 4 | 0.22 | 0.22 | 6.02E-01 | 0.22 | 6.44E-01 | 0.28 |
| rs2235498 | 33238408 | 4 | 0.23 | 0.23 | 2.58E-01 | 0.21 | 2.98E-01 | 0.20 |
| rs9368758 | 33245999 | 4 | 0.07 | 0.07 | 5.92E-01 | 0.07 | 8.49E-01 | 0.05 |
| rs9277932 | 33249231 | 4 | 0.46 | 0.46 | 9.20E-01 | 0.48 | 9.67E-01 | 0.47 |
| rs2855430 | 33249258 | 4 | 0.14 | 0.14 | 5.47E-02 | 0.13 | 5.39E-01 | 0.19 |
| rs3762013 | 33250517 | 4 | 0.46 | 0.46 | 8.91E-01 | 0.48 | 9.32E-01 | 0.47 |
| rs2855425 | 33252351 | 4 | 0.29 | 0.29 | 1.36E-01 | 0.31 | 2.58E-01 | 0.24 |
| rs2855459 | 33262634 | 4 | 0.14 | 0.14 | 6.51E-02 | 0.13 | 5.12E-01 | 0.19 |
| rs2269346 | 33266876 | 4 | 0.07 | 0.07 | 6.05E-01 | 0.07 | 8.45E-01 | 0.05 |
| rs6531 | 33271429 | 4 | 0.29 | 0.29 | 5.32E-02 | 0.32 | 2.59E-01 | 0.24 |
| rs439205 | 33281820 | 4 | 0.24 | 0.25 | 1.11E-01 | 0.22 | 5.38E-01 | 0.27 |
| rs421446 | 33282761 | 4 | 0.27 | 0.27 | 1.47E-01 | 0.24 | 5.19E-01 | 0.27 |
| rs2854028 | 33287667 | 4 | 0.24 | 0.23 | 3.74E-01 | 0.24 | 9.21E-01 | 0.22 |
| rs213213 | 33291708 | 4 | 0.30 | 0.30 | 2.72E-01 | 0.33 | 3.29E-02 | 0.23 |
| rs213212 | 33293896 | 4 | 0.28 | 0.28 | 6.28E-02 | 0.31 | 4.31E-01 | 0.24 |
| rs213220 | 33310618 |  | 0.49 | 0.48 | 7.57E-01 | 0.51 | 8.63E-01 | 0.50 |
| rs213199 | 33343733 |  | 0.34 | 0.35 | 9.58E-01 | 0.32 | 3.48E-01 | 0.33 |
| rs464921 | 33348484 |  | 0.16 | 0.16 | 7.08E-01 | 0.15 | 1.61E-01 | 0.16 |
