# Supplementary material for: Differences in Meiotic Recombination Rates in Childhood Acute Lymphoblastic Leukemia at an MHC Class II Hotspot Close to Disease Associated Haplotypes
Source: PLoS One. 2014 Jun 24;9(6):e100480. doi: 10.1371/journal.pone.0100480 (PMC4069019; doi:10.1371/journal.pone.0100480)
Supplement: Table S2 — Haplotype Numbers Predicted by PHASE. Average numbers of predicted haploytpes using matched numbers of cases and controls (BCP-ALL, 447; T-ALL, 44) based on output data from four replicate PHASE analyses using different seed values. (DOCX) [file pone.0100480.s003.docx]

**Table S2.** **Haplotype Numbers Predicted by PHASE**

| Phenotype | n | Average predicted number of haplotypes | SD |
| --- | --- | --- | --- |
| T-ALL | 44 | 249.5 | 8.38 |
| Controls | 44 | 527.2 | 31.20 |
| BCP-ALL | 447 | 1040 | 22.96 |
| Controls | 447 | 991.25 | 25.62 |
