# Supplementary material for: Differences in Meiotic Recombination Rates in Childhood Acute Lymphoblastic Leukemia at an MHC Class II Hotspot Close to Disease Associated Haplotypes
Source: PLoS One. 2014 Jun 24;9(6):e100480. doi: 10.1371/journal.pone.0100480 (PMC4069019; doi:10.1371/journal.pone.0100480)
Supplement: Table S3 — Tagger Selected SNPs. Thirty five SNPs, selected using Tagger [44] for haplotype analysis with LDsplit [28], showing chromosomal location and minor allele frequency (MAF) in the CEU population. (DOCX) [file pone.0100480.s004.docx]

**Table S3.** **Tagger Selected SNPs**

| SNP rsID | Chr6: (GRCh36) | MAF (CEU) |
| --- | --- | --- |
| rs3135034 | 33059640 | 0.08 |
| rs206776 | 33061689 | 0.28 |
| rs188245 | 33063954 | 0.57 |
| rs3129305 | 33067158 | 0.09 |
| rs206769 | 33069082 | 0.21 |
| rs9500927 | 33069339 | 0.09 |
| rs206768 | 33069365 | 0.43 |
| rs172275 | 33069599 | 0.55 |
| rs206767 | 33070398 | 0.48 |
| rs9276964 | 33072945 | 0.50 |
| rs3128947 | 33073040 | 0.50 |
| rs12216336 | 33075719 | 0.50 |
| rs2894311 | 33076317 | 0.08 |
| rs12191230 | 33076576 | 0.50 |
| rs206762 | 33078428 | 0.46 |
| rs3128931 | 33079686 | 0.21 |
| rs376892 | 33080865 | 0.26 |
| rs3129304 | 33081721 | 0.16 |
| rs2581 | 33082379 | 0.45 |
| rs2582 | 33082529 | 0.12 |
| rs453779 | 33083359 | 0.56 |
| rs2267647 | 33083489 | 0.31 |
| rs375256 | 33083847 | 0.26 |
| rs2284191 | 33084632 | 0.09 |
| rs403414 | 33085293 | 0.17 |
| rs396090 | 33085513 | 0.47 |
| rs6911639 | 33086156 | 0.20 |
| rs429916 | 33086565 | 0.07 |
| rs6457699 | 33089625 | 0.57 |
| rs34570277 | 33089995 | 0.08 |
| rs9276994 | 33092233 | 0.34 |
| rs6936620 | 33092429 | 0.45 |
| rs1367731 | 33093177 | 0.17 |
| rs423639 | 33095752 | 0.08 |
| rs9296068 | 33096673 | 0.31 |
